# Interrogating the sepsis host immune response using cytomics

**DOI:** 10.1186/s13054-023-04366-0

**Published:** 2023-03-21

**Authors:** Robert B. Lindell, Nuala J. Meyer

**Affiliations:** 1 Division of Critical Care Medicine, Department of Anesthesia and Critical Care Medicine, Children’s Hospital of Philadelphia, University of Pennsylvania Perelman School of Medicine, Philadelphia, PA USA; 2 Institute for Immunology, University of Pennsylvania Perelman School of Medicine, Philadelphia, PA USA; 3 Pediatric Sepsis Program, Children’s Hospital of Philadelphia, Philadelphia, PA USA; 4 Division of Pulmonary, Allergy, and Critical Care Medicine, University of Pennsylvania Perelman School of Medicine, Philadelphia, PA USA; 5 Center for Translational Lung Biology and Lung Biology Institute, University of Pennsylvania Perelman School of Medicine, Philadelphia, PA USA

## Abstract

This article is one of ten reviews selected from the Annual Update in Intensive Care and Emergency Medicine 2023. Other selected articles can be found online at https://www.biomedcentral.com/collections/annualupdate2023. Further information about the Annual Update in Intensive Care and Emergency Medicine is available from https://link.springer.com/bookseries/8901.

## Introduction

Sepsis, the syndrome of life-threatening organ dysfunction due to infection, affects an estimated 48 million people annually around the globe and is the most common cause of death in hospitals [1, 2]. Although sepsis is defined as a dysregulated host response to infection [3], our ability to discriminate adaptive and maladaptive immune response is limited. In most serious infections, there exists a complex interplay between pathogen-induced tissue injury, pathogen-directed host inflammation, injury resulting from host immune activation, and potential secondary infections due to impaired or exhausted immune defense. Simultaneous exuberant innate immune activation and hypofunctioning adaptive immune processes are frequently detected in septic individuals [4], and we lack clinical tools to quantify the balance between hyper- and hypo-inflammation [5]. In addition, we cannot confidently ascertain which responses are necessary for microbial control and which may propagate organ injury without influencing pathogen killing. This complexity, and the demonstrated heterogeneity in patient immune response, has contributed to a major void in specific pharmacotherapy for sepsis and prompted calls for immunophenotyping that might allow more precise therapeutic targeting [6]. 

Circulating blood cells offer a unique window to the immune response and reflect both innate and adaptive immune programs. They are relatively easy to obtain and suitable for repeat sampling, thus their trajectories can manifest the dynamic and evolving host response to infection and inflammation. Flow cytometry is the tech- nique of measuring single cells within a suspension by directing a laser or light source at the fluid stream and separating cells by their physical properties such as size and intracellular complexity (granularity), along with fluorescence from chemicals in the cells themselves or from fluorescent-conjugated antibodies to cellular antigens. Detectors capture the light and fluorescence emitted from cells, and the combination of light scatter and fluorescence categorizes cells with uniform size, scatter, and fluorescent features. Whereas early cytometers had only a single laser, modern cytometers combined 4, then 7, then 14 and higher numbers of lasers to facilitate characterization of more than 40 features [7]. This revolution in flow cytometry throughput in turn catalyzed an explosive growth in cellular identification, improving recognition of immune cells and tumor cells in increasing detail. A technical limitation of flow cytometry has been the spectral overlap between fluorophores, which could limit precise identification or constrain the dyes used together. In response, investigators developed mass spectrometry or cytometry by time-of-flight (CyTOF), a technique which replaces fluorescent antibodies with heavy metal isotopes that are not naturally occurring and that have unique mass spectrometry characteristics [7]. Approximately 60 heavy metal isotopes to date have sufficient purity and antibody conjugation chemistry to be studied in a single panel, greatly expanding the dimensionality of cellular characterization [7]. Although CyTOF applications in critical illness are still relatively infrequent [8], we expect applications to grow exponentially as the technology matures and costs decrease. 

Peripheral blood profiling has limitations including the compartmentalization of immune reactions [9] and the inability of circulating cells to capture tissue-resident immune processes. Nonetheless, understanding which immune cells participate in the host response during sepsis may elucidate a clearer picture of regulated and dysregulated host response. In this chapter, we highlight the knowledge attained by cytometric profiling during adult and pediatric sepsis and propose key future research priorities to best harness this information.

## Historical focus on immature granulocytes in sepsis

Long before the molecular era of medicine, hematopathologists had described the association between numeric and morphologic changes in peripheral blood leukocytes and severe infections across the age spectrum. Elevated peripheral white blood cells and a shift to more immature neutrophils or “bands” were codified as part of the systemic inflammatory response syndrome (SIRS) criteria, although with acknowledgement that SIRS could occur due to both infectious and non-infectious causes [10] and that sepsis can occur in the absence of SIRS [11]. As early as the 1970s, investigators had demonstrated that hospitalized patients with infection, both pediatric and adult, were more likely to have immature neutrophils, toxic granulation within neutrophils, and vacuolization of neutrophil cytoplasm on blood smear review [12]. Furthermore, when tested ex vivo, neutrophils from subjects with such morphological changes were more likely to have delayed bacterial killing and higher proportion of neutrophils with visible intracellular bacteria [12], linking altered leukocyte morphology to impaired pathogen response. Along with the discovery of inflammatory cytokines and chemokines that were highly expressed in the circulation of septic patients, a paradigm emerged that posited that sepsis represented unrivaled and uncontrolled inflammation [13]. 

With the rapid advances of flow cytometric techniques, applications to the granulocyte fraction highlighted the shift to more immature neutrophil forms and significant heterogeneity among circulating leukocytes in sepsis. Neutrophils from hospitalized patients with sepsis were more likely to have reduced CD10 and CD16 expression compared to either uninfected outpatients or to patients with community-acquired infection but without SIRS, and the manual band count was correlated with CD10^dim^ CD16^dim^ neutrophils [14]. The antigen CD10 is not restricted to granulocytes—indeed, it is also known as acute lymphoblastic lymphoma antigen—but neutrophils express this antigen only in the latest stages of differentiation [15]. In addition, low or absent CD10 expression was shown to discriminate immature neutrophils in a study of donors treated with exogenous granulocyte colony stimulating factor (G-CSF), the growth factor most known for stimulating neutrophil production from the bone marrow. In contrast to mature CD10^+^ neutrophils, CD10^−^ neutrophils exhibited immunostimulatory effects on CD4^+^ and CD8^+^ T cells, enhancing T cell proliferation, survival, and interferon-gamma (IFNγ) production [15]. Neutrophils with CD10^dim^ CD16^dim^ expression persisted at 3 and 8 days after the onset of septic shock and non-survivors had a higher proportion of these cells compared to septic shock survivors [16]. In addition, neutrophils from patients with septic shock manifested altered function, with lower intracellular myeloperoxidase and lactoferrin expression, reduced chemotaxis, and impaired phagocytosis [16], suggesting that immune dysregulation in sepsis involves both immune stimulation and defective immune functions.

More recently, a class of granulocytes that has a density closer to monocytes, and thus separates with the peripheral blood mononuclear cell fraction when using density gradient separation, has been described. These ‘low density neutrophils (LDN)’ express markers that classically identify granulocyte origin (CD15) and suppress T cell proliferation through the elaboration of arginase which downregulates the T cell receptor zeta-chain expression [17, 18]. Given their suppressive effect on T cell effector function, these cells are known as granulocytic myeloid derived suppressor cells (gMDSC), and they seem to be especially upregulated in sepsis compared to comparably critically ill controls [18]. In addition, gMDSC express high levels of arginase-1 and neutrophil degranulation markers, many of which contribute to a transcriptomic phenotype corresponding to plasma protein hyperinflammation during acute respiratory distress syndrome (ARDS) [19]. Thus, gMDSC/LDN are a unique class of cells that may contribute to both arms of the dysregulated immune response in sepsis, innate inflammation with neutrophil degranulation, and suppression of T cell effector function.

## Compensatory anti-inflammatory response and sepsis immune paresis

The past 25 years have witnessed an increasing focus on the downregulated aspects of immune function during sepsis, sometimes termed sepsis immune paresis or the compensatory anti-inflammatory response [13, 20]. Building from animal studies in which significant lymphoid apoptosis was observed in models of uncontrolled infection, investigators leveraged rapid autopsy studies to demonstrate that septic patients frequently had at least focal apoptosis in the spleen and colon [21]. Striking lymphopenia was often observed in patients with sepsis, and persistent lymphopenia beyond day 4 predicted a higher risk of death [22]. Applying flow cytometry, studies demonstrated that exogenous endotoxin, which typically stimulates an increased density of human leukocyte antigen-DR isotype (HLA-DR), a ligand for the T-cell receptor, on monocytes, failed to stimulate monocyte HLA-DR (mHLA-DR) expression in patients with sepsis or following severe trauma [23]. Elegant studies used endotoxin to stimulate ex vivo peripheral white blood cells collected from patients with sepsis and demonstrated that the monocytes lacking HLA-DR were also deficient in antigen-presenting capacity and in producing inflammatory cytokines, such as tumor necrosis factor alpha (TNF-α), in response to endotoxin [24]. In many ways, the monocytes from human subjects with sepsis seemed to resemble cells that had been desensitized with repeated doses of endotoxin [24], and CD14-expressing (monocytic) cells with HLA-DR^dim^ expression are sometimes termed monocytic MDSC.

Monneret and colleagues profiled more than 90 subjects with septic shock and observed that whereas the proportion of low mHLA-DR cells was similar between survivors and non-survivors at admission, the persistent expression of fewer than 30% mHLA-DR^+^ cells after 48 h was strongly associated with mortality [25]. Since this pivotal early work establishing mHLA-DR as a potential marker for sepsis immunosuppression, it has remained a strong candidate to identify subjects in real time with immune deficits. In an early phase 2 precision medicine sepsis trial, subjects with sepsis and confirmed low mHLA-DR were randomized to placebo or a daily dose of granulocyte–macrophage colony-stimulating factor (GM-CSF) for 8 days. Subjects randomized to GM-CSF restored their mHLA-DR expression and in vitro*/*ex vivo endotoxin-stimulated cytokine production [26]. GM-CSF-treated subjects also had improved severity of illness scores and shorter duration of mechanical ventilation, though the trial was not powered for clinical outcomes [26]. Larger trials in unselected patients with acute respiratory failure did not reproduce these findings [27], leaving many to wonder whether limiting the drug to those with low mHLA-DR might have been more effective. The expression of mHLA-DR remains a strong candidate for identifying sepsis immunosuppression in real time and newer trials may use this marker as a criterion for entry.

## Lymphocyte activation and exhaustion

The past 5 years have brought more of an appreciation that rather than a compensatory anti-inflammatory response following immune hyperactivity, both hyperactivation and immunosuppression occur simultaneously, across different cell types and in different compartments [4, 9]. A focused interrogation of the B lymphocyte fraction demonstrated that this population undergoes specific depletion of memory B cells through activation-associated apoptosis pathways [28]. The significance of this finding is that in order to preferentially restore B cell function, a strategy may need to not only restore the number of B-cells but also expand or replenish the memory cell pool, which could necessitate antigen challenge [28].

A potential link between excessive activation and another aspect of lymphocyte dysregulation, exhaustion, is also observed in the T cell fraction. In a prospective study of subjects with sepsis compared to those with non-infectious critical illness, and with healthy controls, the T lymphocytes from patients with sepsis demon- strated increased markers of activation and of exhaustion [29]. Both B- and CD4^+^ T-lymphocytes from patients with sepsis seem to overexpress the exhaustion marker programmed death 1 (PD-1) and its ligand PD-L1 [3], and T-lymphocytes also have been shown to overexpress inhibitory markers such as T-cell immunoglobulin and mucin domain-containing protein-1 (TIM-1), lymphocyte activation gene 3 (LAG-3), and cytotoxic T-lymphocyte-associated protein 4 (CTLA4) [29]. At the same time, populations of hyperactivated, proliferating T cells have been identified in adult and pediatric sepsis [31, 32].

Coronavirus disease 2019 (COVID-19) also focused attention on T cell hyperactivation as a potential marker for more severe immune dysregulation. High dimensional flow cytometric profiling of subjects highlighted considerable heterogeneity in the immune response even among hospitalized patients with COVID-19 [33]. Whereas some patients displayed almost no activation of B- or T-lymphocytes, others exhibited dramatic CD4^+^ and CD8^+^ T lymphocyte activation and proliferation, and the integrated immunotype characterized by this activation was associated with severity of illness, greater need for respiratory support, and higher mortality [33]. Pediatric subjects with multisystem inflammatory syndrome in children (MIS-C) also demonstrated highly activated T-lymphocytes [34], although with particular activation in the ‘vascular patrolling’ CX3CR1^+^ CD8^+^ T cell population that was not observed in hospitalized adult patients with acute COVID-19 [33]. Because the poor prognosis immunotype of acute COVID-19 was dominated by highly activated T cells but also enriched in exhaustion markers including PD-1 [33], there is strong interest in understanding better the relationship between T cell exhaustion and activation.

**Fig. 1:**
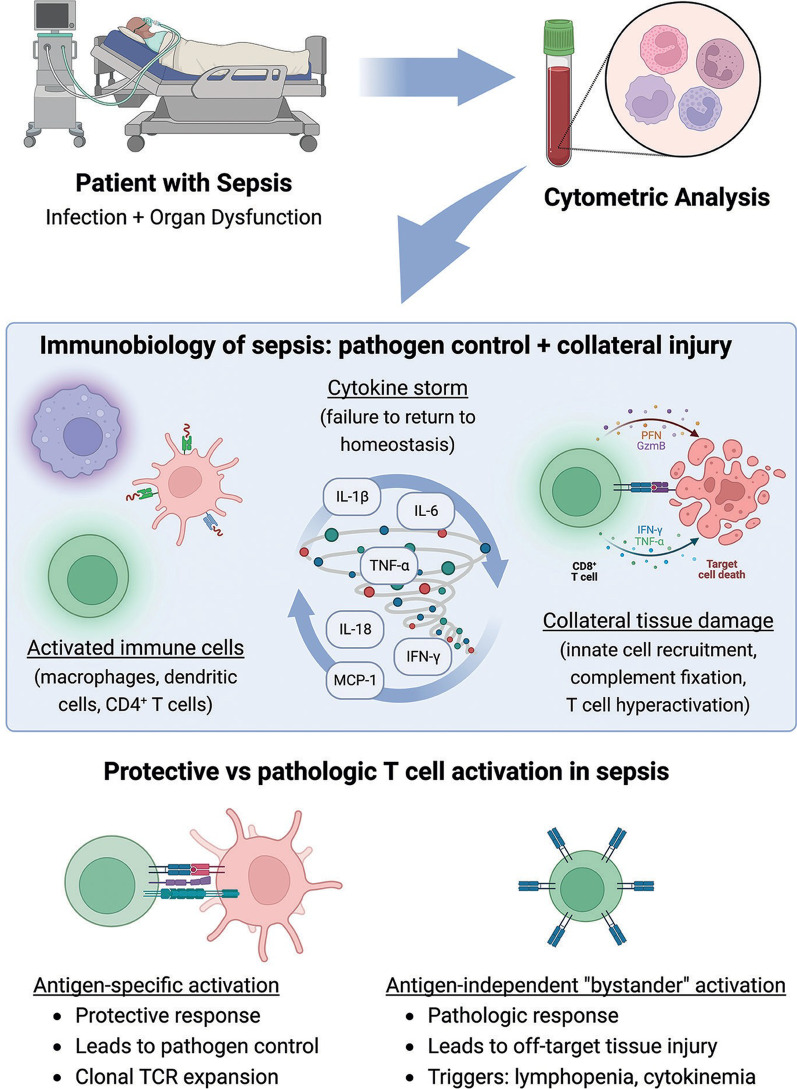
Potential utility of cytometric profiling to understand the sepsis immune response. Peripheral blood leukocytes from patients with sepsis can be assayed by either flow cytometry or mass cytometry (cytometry by time-of-flight [CyTOF]) to understand cell populations and, via their expression patterns, their degree of activation or exhaustion. Along with plasma protein analysis which could inform about cytokine elaboration, immune cell profiling might detect the source of inflammatory proteins and identify specific deleterious patterns. Cytotoxic T cell activation, which is associated with poor outcomes in sepsis and COVID-19 and contributes to tissue damage, may be due to antigen-driven processes that are necessary for pathogen control, or it may result from bystander activation. Discriminating between these patterns may be essential to best design strategies to intervene in pathologic activation yet preserve patho- gen clearance. *TNF* tumor necrosis factor, *IL* interleukin, *IFN* interferon, *MCP* monocyte chemoattrac- tant protein, *PFN* perforin, *GzmB* granzyme B, *TCR* T-cell receptor. Figure created with BioRender.com

The mechanism by which these critically ill patients develop T cell hyperactivation is unknown, which limits our ability to deploy precision immunotherapeutics for these patients. Early in critical illness, T cell hyperactivation can occur via two distinct mechanisms: antigen-dependent activation via T cell receptor signaling [35], or a dysregulated, antigen-independent ‘bystander’ activation [33], as shown in Fig. 1. In a precision medicine paradigm for sepsis, different approaches to immune modulation could be indicated based on the antigen-specificity of the T cell response. Antigen-specific hyperactivated responses might improve pathogen clearance, and thus we might attempt to preserve this activation. In contrast, bystander activation might drive off-target tissue injury, and a precision paradigm might try to blunt such activation. If we could use cytometric profiling to distinguish these patterns of T-cell hyperactivation, more precise immunomodulation might be possible.

## Pediatric sepsis: cytomics in the developing immune system

Since pediatric-specific criteria for sepsis were defined in 2005 [37], the burden of sepsis in children has been studied extensively [38, 39]. Although less prevalent than adult sepsis, pediatric sepsis is the leading cause of death of hospitalized children worldwide [1]. Sepsis incidence and outcomes vary dramatically by age and comorbidities, with younger children [39], immunocompromised children [40, 41], and children who develop immune dysfunction in the setting of sepsis [42] representing the highest risk clinical phenotypes.

As in adults, sepsis in children is characterized by concurrent pro- and anti-inflammatory states with dysregulation of the innate and adaptive immune responses to infection [37]. Mirroring translational investigations in adults, pediatric sepsis research has increasingly focused on defining the molecular biology of the disease. Many pediatric patients with sepsis develop innate and adaptive immune dysfunction, which is typically referred to as “immune paralysis” [43, 44] and can be identified by impaired whole blood ex vivo TNF-α and IFNγ production capacity in response to antigen stimulation. Immune paralysis in pediatric sepsis is associated with secondary infection, persistent organ dysfunction [42], and mortality [40, 41, 45]. Mitochondrial dysfunction is a hallmark of this sepsis-associated immune suppression and has been associated with organ failure in both pediatric and adult sepsis [46].

In contrast to these functional assays, cellular and molecular approaches to immune profiling have also been applied to cohorts of pediatric sepsis patients with the goal of identifying sepsis subphenotypes that could be amenable to precision therapy. Using clinical characteristics and candidate biomarkers, investigators have identified three major inflammation subphenotypes in pediatric sepsis patients: immune paralysis (characterized by persistent antigen stimulation, decreased mHLA-DR expression, and decreased cytokine production in the setting of mitogen stimulation), sequential multiple organ failure (characterized by respiratory and liver failure, and oligogenic mutations in FAS/FAS ligand), and thrombocytopenia-associated multiple organ failure (characterized by hemolysis, thrombocytopenia, and oligogenic mutations in complement or ADAMTS13 signaling) [47]. Investigators studying the adaptive immune response in pediatric sepsis have employed unsupervised clustering of bulk transcriptomics data to identify two major subclasses of sepsis driven primarily by differences in T cell and B cell receptor signaling pathways [48]. These subclasses have been shown to have differential response to corticosteroid administration [49], and hospital mortality is substantially increased in the subgroup with downregulated signaling [48]. Finally,investigators have recently employed flow cytometry and metabolomics to demonstrate an association between T cell immunometabolic dysregulation and other markers of immune paralysis in a pilot study of pediatric sepsis patients [31]. In recognition of the association between immune dysfunction and clinical outcomes in pediatric sepsis, the most recent guidelines for monitoring organ dysfunction in pediatric critical illness specifically highlighted the need to develop capabilities for clinical monitoring of immune dysfunction in the pediatric intensive care unit (ICU) [50]. Because the pediatric immune system exhibits remarkable biologic heterogeneity driven by host characteristics, infectious exposures, and pubertal status among other factors, future research searching for a ‘treatable trait’ in pediatric sepsis patients with immune dysregulation will require a detailed understanding of the developing pediatric immune system in both health and disease.

## Conclusion

Sepsis remains a critical threat to the health of adults and children worldwide. In the search for markers of dysregulated host immune reactions to infection, cytometric profiling of circulating leukocytes has yielded potential candidates to identify both hyperactive *and* hypofunctioning immune responses. If precision immunomodulatory approaches are to be successful, validated tools that reliably identify favorable and maladaptive patterns will be essential. Clinical trials are encouraged to collect peripheral blood leukocytes to enable discovery and validation of the most reliable cytometric features. Future research could focus on whether these markers, if determined prospectively, might act as enrichment tools to select patients at high risk for poor outcomes or with a differential therapeutic response.

## Data Availability

Not applicable.
